# A Case of Hepatic Cyst–Induced Internal Jugular Venous Thrombosis

**DOI:** 10.1016/j.case.2021.02.007

**Published:** 2021-04-09

**Authors:** Tuyen V. Le, Thai Truong, Tam N.M. Ngo, Quoc Bui, Cassady Palmer, Vien T. Truong, Duc H. Nguyen

**Affiliations:** a Department of Cardiology, Quang Tri General Hospital, Dong Ha, Vietnam; b Department of Internal Medicine, University of Medicine and Pharmacy at Ho Chi Minh City, Ho Chi Minh City, Vietnam; c Pham Ngoc Thach University of Medicine, Ho Chi Minh City, Vietnam; d Washington University School of Medicine, St. Louis, Missouri; e The Christ Hospital Health Network and Lindner Research Center, Cincinnati, Ohio

**Keywords:** Hepatic cyst, Thrombosis, Cardiac complication

## Abstract

• Echocardiography can demonstrate hepatic cyst–induced right atrial compression.
• Hepatic cyst–induced blood flow stasis can cause internal jugular venous thrombus.
• Laparoscopic deroofing of hepatic cysts is a safe and effective treatment.

## Graphical abstract

**Figure:**
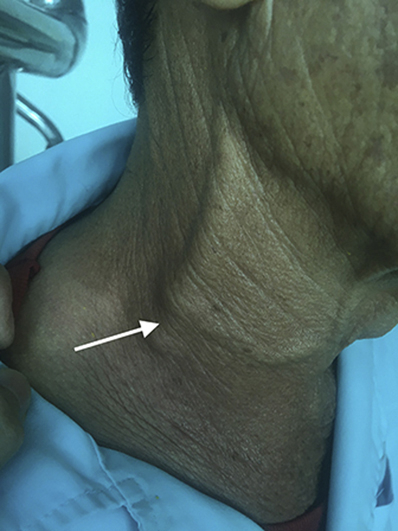

## Introduction

We report a case of hepatic cyst–induced cardiac complication. Multiple hepatic and renal cysts were visualized on abdominal computed tomography in a 69-year-old male patient. One of the massive hepatic cysts was seen compressing the right atrium, leading to superior vena cava (SVC) syndrome and right internal jugular venous thrombi. The patient presented with headache and right-sided neck and bilateral upper extremity edema for 1 month. These symptoms diminished following hepatic cyst deroofing accompanied by long-term anticoagulant therapy. To our knowledge, this is the first case report of SVC syndrome and right internal jugular venous thrombi that resulted from hepatic cyst–induced blood flow stasis.

## Case Presentation

**Figure 1:**
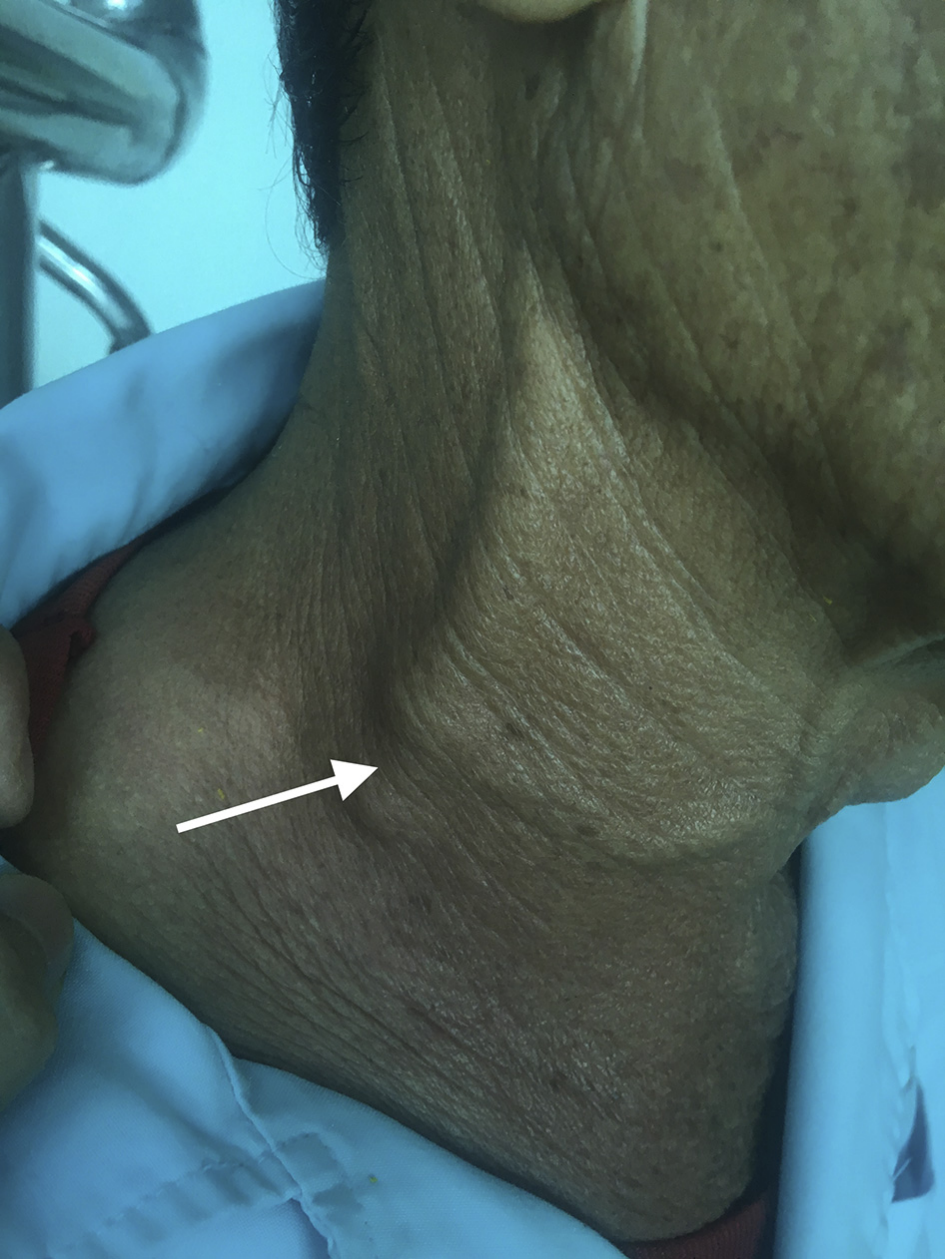
Swelling along the anterior border of right sternocleidomastoid muscle and distension of the right jugular vein (*arrow*).

A 69-year-old man was admitted to the hospital for right-sided neck and bilateral upper extremity edema and headache for 1 month. Vital signs showed a heart rate of 70 beats/min, arterial blood pressure of 120/70 mm Hg, a respiratory rate of 20 breaths/min, and body temperature of 37°C. Physical examination revealed swelling along the anterior border of the right sternocleidomastoid muscle and distension of the right jugular vein (Figure 1). Chest and cardiac physical examination were unremarkable. He had no history of tobacco or intravenous drug use. Abnormal laboratory findings included a high serum level of total bilirubin (23 μg/L), direct bilirubin (6.2 μg/L), and indirect bilirubin (16.8 μg/L). Results of complete blood count, renal function analysis, liver function analysis, coagulation test, and electrocardiography were within normal limits.

**Figure 2:**
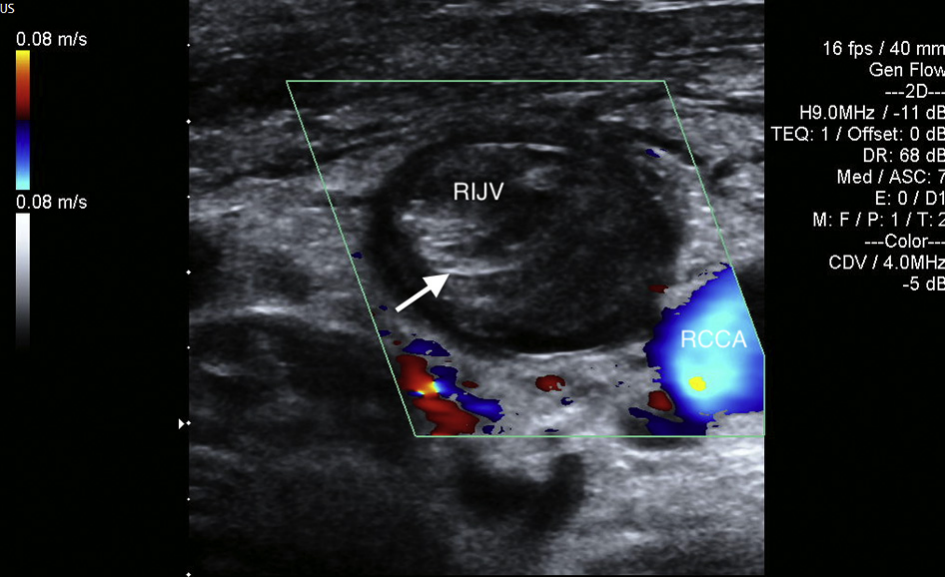
Thrombus visualized within the right internal jugular vein (RIJV; *arrow*). The vessel could not be compressed. *RCCA*, Right common carotid artery.

**Figure 3:**
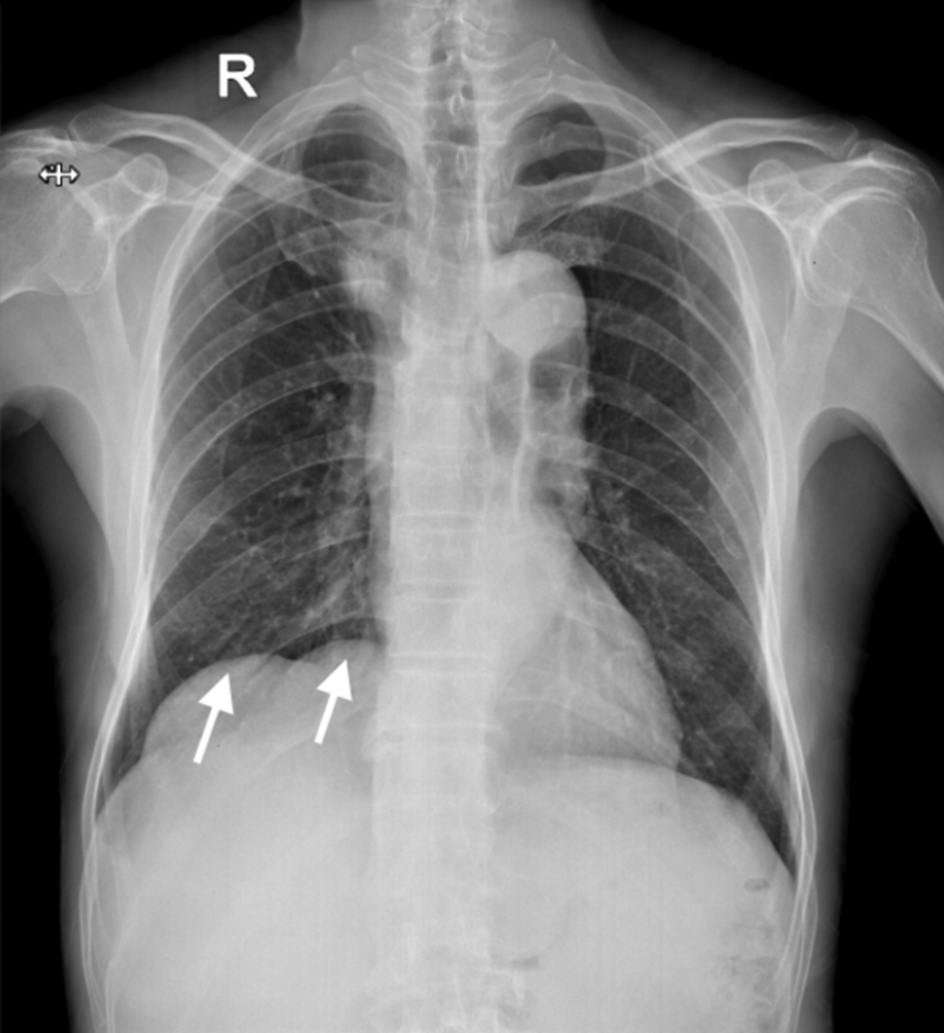
Two dome-shaped elevations of the right hemidiaphragm on the chest radiograph (*arrow*).

**Figure 4:**
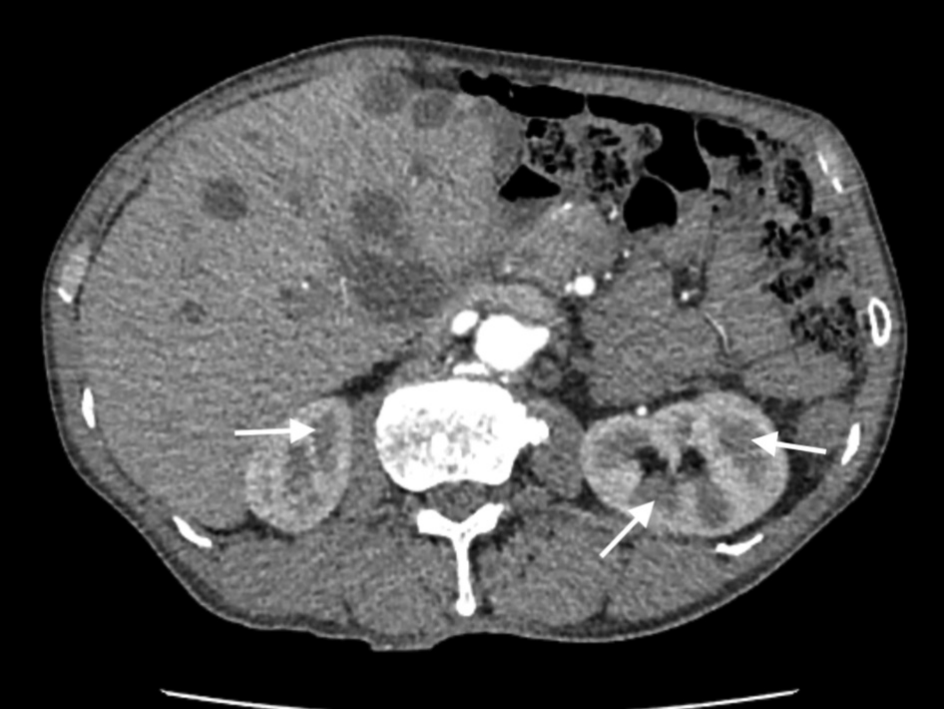
Multiple variable-sized cysts in both kidneys (*arrow*).

**Figure 5:**
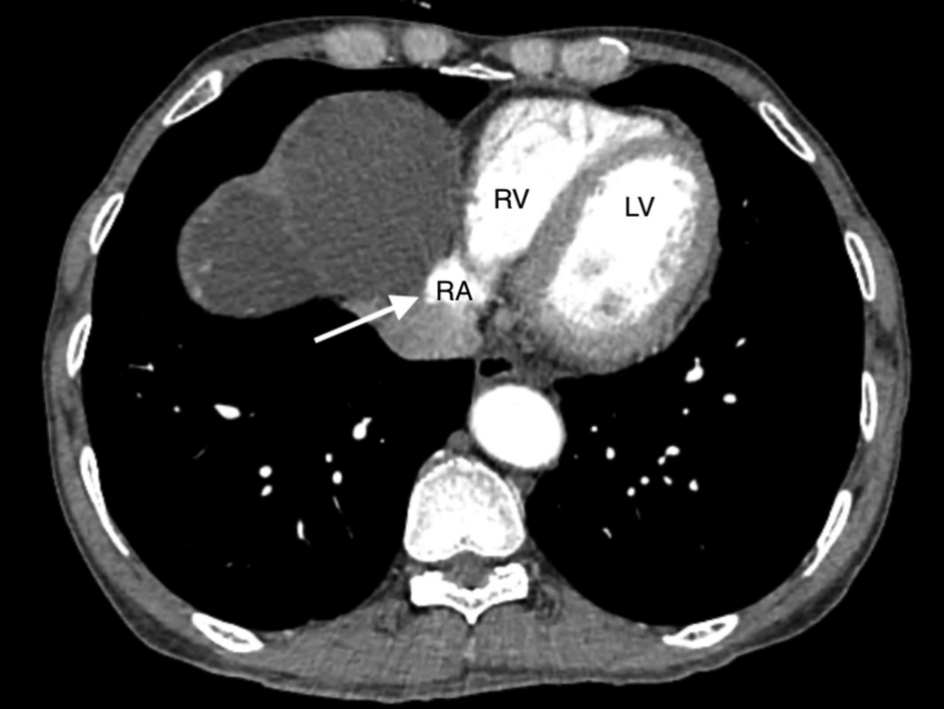
Right atrial compression by multiple massive hepatic cysts on computed tomography (*arrow*).

**Figure 6:**
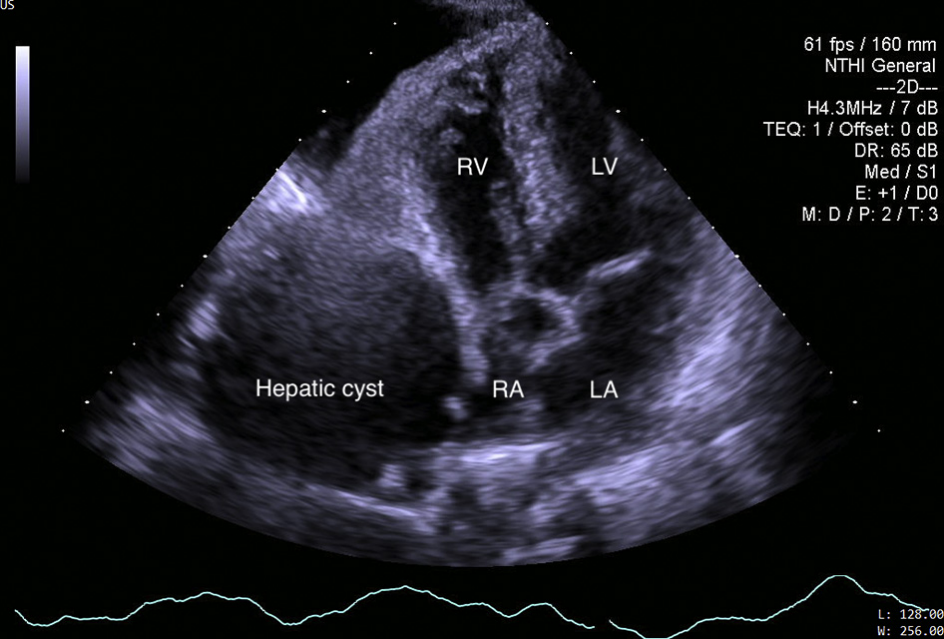
The hepatic cyst compressing the right atrium seen on transthoracic echocardiography.

Carotid ultrasound demonstrated thrombi in the right internal jugular vein (Figure 2). Chest radiography finding revealed two dome-shaped elevations of the right hemidiaphragm suggestive of intra-abdominal masses (Figure 3). Computed tomography showed multiple variable-sized cysts in both kidneys (Figure 4) and the liver and massive hepatic cyst–induced right atrial compression (Figure 5). Echocardiography demonstrated a normal left ventricular ejection fraction with no wall motion abnormalities or valvular dysfunction, and a masslike structure consistent with hepatic cyst was seen compressing the right atrium (Figure 6, Video 1).

**Figure 7:**
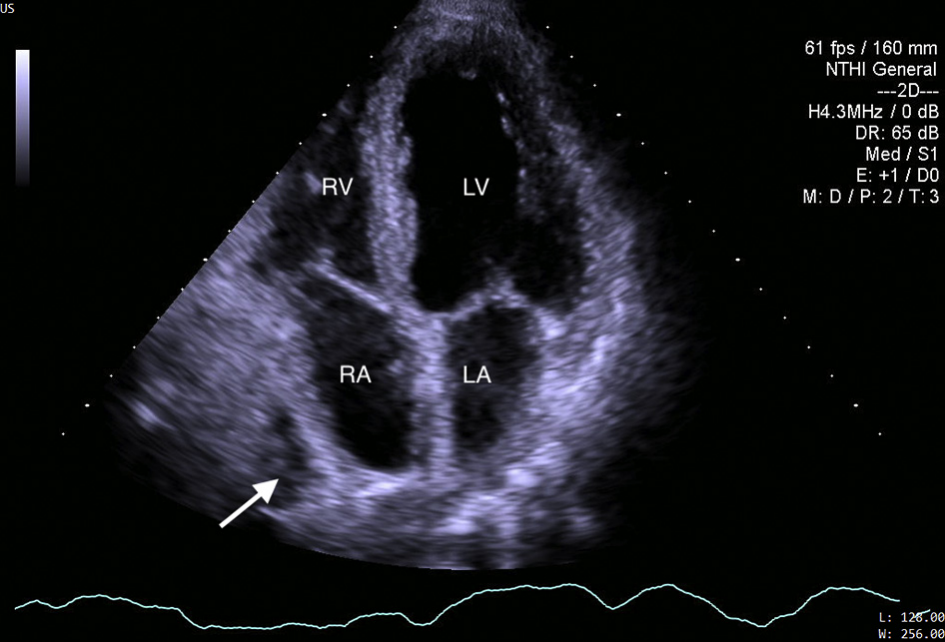
Recovery of right atrial function on postsurgical transthoracic echocardiography. Small hepatic cyst after laparoscopic deroofing (*arrow*).

**Figure 8:**
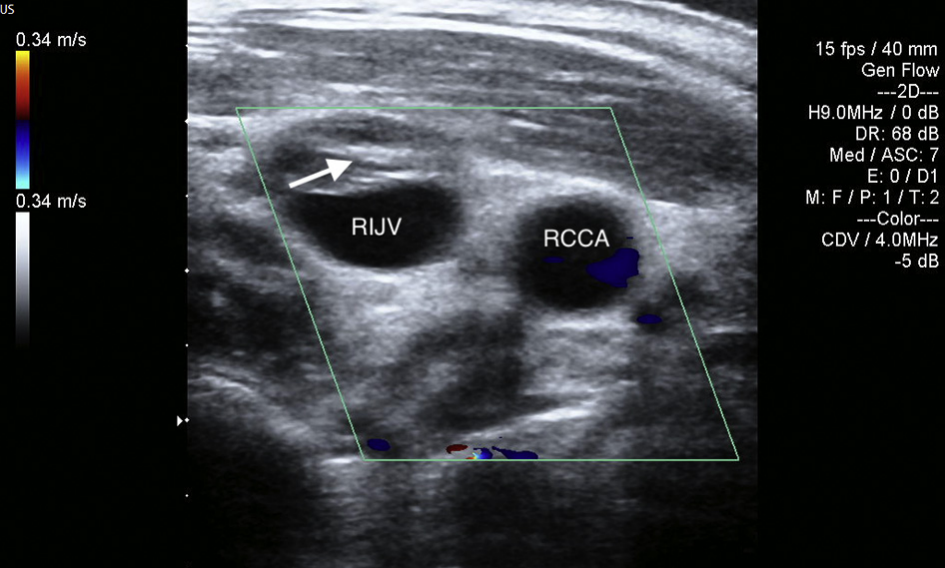
Partial resolution of thrombi in the right internal jugular vein (*arrow*) on day 7 following anticoagulant therapy on postoperative carotid ultrasound.

The patient underwent uncomplicated laparoscopic deroofing of the hepatic cysts. One liter of drainage fluid was obtained from the largest cyst. Gram stain, culture, and cytology of the fluid were negative. Simple liver cysts were lined by cuboidal epithelium and supported by a fibrous wall found on histopathology. The patient was treated with 7 days of subcutaneous low–molecular weight heparin (1 mg/kg every 12 hours) followed by a long-term vitamin K antagonist. Postsurgical transthoracic echocardiography demonstrated elimination of compression and recovery of right atrial function (Figure 7, Video 2). Partial resolution of venous thrombi was seen by carotid ultrasound on day 7 following the anticoagulant therapy (Figure 8). This was accompanied by a significant alleviation of the patient's symptoms.

## Discussion

Hepatic cyst–induced cardiac complications are unusual. The right heart is the most commonly involved site. Because of the anatomic vicinity, hepatic cysts may impinge the right atrium and ventricle, resulting in right heart dysfunction, with dyspnea on exertion as the most common symptom.^1^^,^^2^ Atrial premature beats have also been reported as a consequence of right atrial compression from hepatic cysts.^3^ Our patient presented with peripheral edema and headache, which resulted from the clinical manifestation of SVC syndrome occurring once the blood flow in the SVC was partially or completely obstructed. In addition, the detection of multiple bilateral renal cysts on computed tomography suggested polycystic kidney disease.

The therapeutic strategy for hepatic cysts depends on the presentation of symptoms. It is recommended that asymptomatic cysts be observed only, whereas symptomatic cysts should be managed by laparoscopic deroofing.^4^ A laparoscopic approach is the procedure of choice for accessible cysts because of its safety and efficiency.^5^ Recurrence rate following laparoscopic deroofing of solitary simple cysts is 14.3%.^6^ Potential complications of the procedure include hemorrhage, infection, and brachial vein thrombosis.^5^^,^^6^ Besides laparoscopic deroofing, our patient needed additional therapy for anticoagulation of thrombosis, which led to significant improvement.

## Conclusion

The right heart is the most commonly involved site of hepatic cyst–induced cardiac complications. Clinical manifestations include dyspnea on exertion, cardiac arrhythmia, peripheral edema, and venous thrombosis. Therapeutic strategy for symptomatic hepatic cysts associated with venous thrombosis is laparoscopic deroofing, followed by anticoagulation.

## Supplementary Data

Video 1 Hepatic cyst compressing the right atrium seen on transthoracic echocardiography.

Video 2 Postsurgical transthoracic echocardiography demonstrating recovery of right atrial function and elimination of compression.
